# Age differences in heroin and prescription opioid abuse among enrolees into opioid treatment programs

**DOI:** 10.1186/1747-597X-6-11

**Published:** 2011-06-02

**Authors:** Charles M Cleland, Andrew Rosenblum, Chunki Fong, Carleen Maxwell

**Affiliations:** 1 New York University College of Nursing, 726 Broadway, New York City, New York, USA; 2 Institute for Treatment Services Research, National Development and Research Institutes, 71 West 23rd Street, New York City, NY, USA; 3 American Association for the Treatment of Opioid Dependence, 225 Varick Street, New York City, NY, USA

## Abstract

**Background:**

In the United States, among those entering opioid treatment programs (OTPs), prescription opioid (PO) abusers tend to be younger than heroin users. Admissions of older persons to OTPs have been increasing, and it is important to understand typical patterns of use among those older enrolees.

**Methods:**

To disentangle the effect of age on recent heroin and PO abuse 29,114 enrolees into 85 OTPs were surveyed across 34 states from 2005-2009. OTPs where PO use was prevalent were oversampled.

**Results:**

Mean age was 34; 28% used heroin only. Younger enrolees had increased odds of using POs relative to using heroin only but mixed model analysis showed that much of the total variability in type of use was attributed to variation in age between OTPs rather than within OTPs.

**Conclusions:**

Organizational and cultural phenomena (e.g., OTP characteristics) must be examined to better understand the context of individual characteristics (e.g., age). If nesting of enrolees within OTPs is ignored, then associations that primarily operate at the OTP level may be misinterpreted as exclusively dependent on individuals.

## Background

Based on the National Survey on Drug Use and Health conducted by the Substance Abuse and Mental Health Services Administration (SAMHSA), misuse of opioids is prevalent in the U.S. with 4.7 million adolescents or adults misusing prescription opioids (POs) in the past month in 2008, which is about 2% of the total U.S. population of adolescents and adults [1]. The prevalence of past month use of heroin in 2008 was 0.1% among persons aged twelve and older. Between 1997 and 2007, approximately 15% of all substance abuse treatment admissions identified heroin as the primary substance of abuse; admissions identifying other opiates as the primary substance of abuse increased from about 1% in 1997 to 5% in 2007. Substance abuse treatment entry data for 2007 suggest users whose primary substance of abuse was heroin were somewhat older than users whose primary substance of abuse was an opioid other than heroin.

Several studies have documented differences between opioid treatment program (OTP) patients who were primarily prescription opioid (PO) abusers versus those where primarily heroin users [2-4]. Among these findings are that heroin users compared with PO abusers are older, report recent income from illegal sources, injection, greater quantity of use, and more family/social problems. These results suggest that, among those entering treatment, PO abusers tend to be younger than heroin users.

Based on Treatment Episode Dataset (TEDS) data, there has been a substantial increase, for the past two decades, in treatment admissions for opioid abuse [5]. Among first-time treatment admissions there has also been a change in the pattern of substance use among older enrolees represented by an increasing illicit drug involvement, e.g. cocaine and heroin [6]. A closer examination of the TEDS data shows that between 1998 and 2008, substance abuse treatment admissions for problematic opioid abuse in the U.S. (heroin and POs) increased 51% [5]. For adolescents and young adults between the ages of 12-25, the increases in treatment admissions are particularly startling: 69% for heroin and about a twenty-fold increase (1,896%) for POs. Differential increases in treatment admissions (heroin for older persons and POs for younger persons) suggest that it is important investigate type of opioid use by age cohort among enrolees into OTPs.

The objectives of the current study were to determine the relationship between three different types of opioid use (heroin only, POs only, both heroin and POs) among OTP enrolees and the age of individual enrolees. The data collection protocol for this study was conceived and executed under the auspices of the Researched Abuse, Diversion and Addiction-Related Surveillance (RADARS^®^) System [7].

## Methods

### Sample

Data were collected from January 2005 through December 2009 from 36,928 enrolees in 85 OTPs located in 34 states. Not all programs began study participation in January of 2005; 70 (82%) OTPs participated for 2 or more years; 12 (14%) participated for at least one year but less than two years; and 3 (4%) for less than one year. All programs were federally approved opioid agonist treatment programs and followed federal methadone treatment protocols that require an opioid-dependence diagnosis and an addiction history of at least one year [8]. The research protocol was approved by the Institutional Review Board of the National Development and Research Institutes, Inc., and oral informed consent was obtained from the study participants. The respondents in this study include treatment seeking persons who reported abusing POs or heroin in the past 30 days and were not in methadone treatment in the previous 30 days. Details of OTP participation and subject recruitment protocol can be found in an earlier publication by our group [4] and are only briefly reviewed here.

OTPs were selected based on our knowledge on which OTPs would most likely participate in the study and to represent regions in the U.S. where prescription opioid (PO) abuse was believed to be prevalent, e.g., non-urban areas, especially those in the Appalachian region. Some OTPs were located in major metropolitan areas such as San Francisco and New York City, where PO abuse among OTP enrolees is believed to be relatively less prevalent. A total of 7,814 respondents were not included in analysis due to missing data on age (n = 1037), age less than 16 years (n = 5), age greater than 70 years (n = 24), missing data on type of opioid use in the month before enrolment (n = 1713), or use of neither heroin nor POs in the month before enrolment (n = 5271), resulting in a sample for analysis of 29,114 OTP enrolees.

### Measures

#### Age

Enrolees were asked to indicate their age in whole years. For summary and analysis, seven age categories were formed: 16-19; 20-25; 26-29; 30-39; 40-49; 50-59; and 60-70. In analysis to determine associations between enrolee age and the probability of each type of opioid use, Helmert contrasts were applied to these age categories so that each age group except for the first was compared with all younger enrolees. The mean age of patients in each OTP and deviations of individual enrolee age from the OTP mean age were calculated.

#### United States region

OTPs were classified as falling into one of four U.S. regions. These regions, as designated by the U. S. Census Bureau [9], are: **Northeast **(Maine, New Hampshire, Vermont, Massachusetts, Rhode Island, Connecticut, New York, New Jersey, Pennsylvania); **Midwest **(Ohio, Indiana, Illinois, Michigan, Wisconsin, Minnesota, Iowa, Missouri, North Dakota, South Dakota, Nebraska, Kansas); **South **(Delaware, Maryland, District of Columbia, Virginia, West Virginia, North Carolina, South Carolina, Georgia, Florida, Kentucky, Tennessee, Alabama, Mississippi, Arkansas, Louisiana, Oklahoma, Texas); and **West **(Montana, Idaho, Wyoming, Colorado, New Mexico, Arizona, Utah, Nevada, Washington, Oregon, California, Alaska, Hawaii).

#### Beale urbanicity code

OTPs were in counties coded as high density areas (population > 1 million); moderately populated counties (≥250,000 and <1 million residents), and low populated counties (<250,000 residents); these three categories were determined by a modified version of the Beale Urbanicity Code [10].

#### Recent heroin use

Enrolees were asked whether they had used heroin in the 30 day period before admission (Yes/No).

#### Recent prescription opioid use

Enrolees were asked whether they had used a prescription opioid to get high in the 30 day period before admission (Yes/No).

### Data Analysis

To account for clustering of the 29,114 patients in 85 OTPs, multinomial logit mixed model analysis with a random intercept [11] was used to predict 1) heroin use only; 2) PO abuse only; and 3) both types of use for individual patients. The *xtlogit *and *gllamm *procedures of the Stata program [12,13] were used to fit these mixed models. In all multinomial logit mixed models, the reference category for the dependent variable of type of opioid use was heroin only. Patient-level predictors included Helmert contrasts of the seven age groups (where each contrast compares one age cohort with all younger respondents) and contrasts of interview year (2005-2009) where the initial year of the survey, 2005, was the reference category. Program-level predictors included urbanicity, U.S. region, and the program average of the age Helmert contrasts. Including the program average of individual age group contrasts disentangles the effects of individual patient age and the effects of the average age of patients in an OTP [14]. While the program average of the contrasts are not of direct interest, including them allows the enrolee-level age contrasts to be interpreted unambiguously as pooled within-OTP regression coefficients. This approach of breaking down the age predictor into within-OTP and between-OTP components is used to estimate the effects of individual age within OTPs [15]. Point-biserial correlations between age and type of opioid use were estimated using the within and between analysis (WABA) approach [16]. The WABA approach decomposes associations between age and type of opioid use into within-OTP and between-OTP components. The Mplus modelling software [17] was used to estimate covariances between age and type of opioid use and variances at each level. These covariances were tested for significance using z-tests and converted to correlations for presentation.

## Results

Table 1 presents data on the characteristics of enrolees and treatment programs. More than half of all programs (53%) were located in densely populated urban areas, and at least fourteen programs were sampled from each U.S. region. In 2008 there were more than 1200 OTPs in the US with 381 in the Northeast, 391 in the Southeast, 183 in the Midwest, and 265 in the West [18]. Regionally, the respective distribution of study OTPs and all OTPs was: Northeast (25% and 31%); Southeast (39% and 32%); Midwest (17% and 15%); West (20% and 22%). PO use only (43%) was the most common type of use in the month before OTP enrolment, but a substantial number of patients used only heroin (28%) or both heroin and POs (29%) in the month before OTP enrolment. Mean age of individual enrolees was 34 years (median = 32; SD = 10.6; min = 16; max = 70). The mean of the average age of enrolees in each OTP was 34 years (median = 34; SD = 4.2; min = 27; max = 46). Among OTP enrolees in the youngest cohort, 1% were 16, 2% were 17, 32% were 18, and 66% were 19. The standard deviation of age in each OTP ranged from 4.8 to 15.1, and the difference between the oldest and youngest enrolee in each OTP ranged from 21 to 53 years (median = 43). The intraclass correlation for age was = .140 [95% CI: 0.105 - 0.182]. This indicates variance in age within programs was substantial.

**Table 1:** Opioid Treatment Program and Enrolee Characteristics

	N	%
**Program Variables (n = 85)**		
U.S. Region		
*Northeast*	21	24.71
*Southeast*	33	38.82
*Midwest*	14	16.47
*West*	17	20.00
Beale Urbanicity		
*Metro Area > 1 million*	45	52.94
*≥ 250k and < 1 million*	25	29.41
*< 250k*	15	17.65
**Enrolee Variables (n = 29114)**		
Age		
*16-19*	679	2.33
*20-25*	6614	22.72
*26-29*	5074	17.43
*30-49*	7867	27.02
*40-49*	5760	19.78
*50-59*	2807	9.64
*60-70*	313	1.08
Interview Year^†^		
2005	6387	21.94
2006	6502	22.33
2007	4669	16.04
2008	5566	19.12
2009	5983	20.55
Prescription Only Past 30 Days	12495	42.92
Heroin Only Past 30 Days	8024	27.56
Prescription & Heroin Past 30 Days	8595	29.52

^† ^Interview year was missing for seven enrolees.

**Figure 1:**
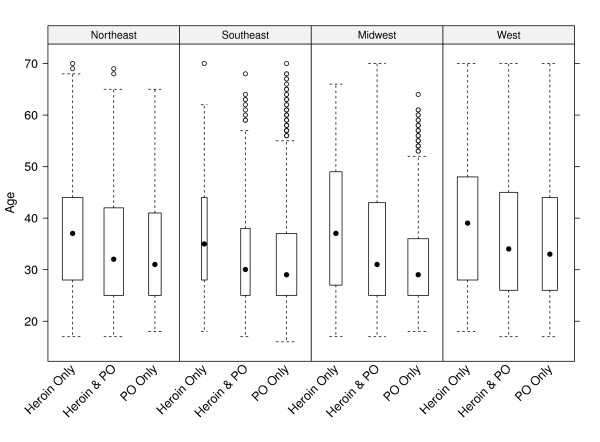
**Age Distribution by Region and Type of Opioid use**.

Figure 1 shows the distribution of enrolee age within each U.S. region and by type of opioid use in the month before OTP enrolment. The dot in the center of each of these boxplots is the median age, while the boxes mark the middle 50% of each age distribution. Unusual observations within each age distribution are plotted individually with open circles. The width of each boxplot is in proportion to the number of enrolees in each group. These boxplots show that, in each U.S. region, enrolees using only heroin in the month before OTP enrolment were approximately five years older than enrolees using either POs only or both POs and heroin. (When region is not considered, differences among median ages are somewhat larger; PO only 30, heroin only 37, both 31.) These boxplots also show that individual enrolees of all ages in the range studied appear in each combination of U.S. region and opioid use in the month before OTP enrolment.

Variability in opioid use across treatment programs. Prior to the focus on differences in opioid use associated with individual age, we explored program variability in opioid use. For each pattern of use, a multilevel logistic regression was fit using *xtlogit *of Stata to estimate the intraclass correlation and the median odds ratio [19]. These are both ways of quantifying variability in type of use associated with OTPs. Interval estimates (95%) for intraclass correlations were .44 - .61, .40 - .55, and .16 - .26 for heroin only, POs only, and both, respectively. These intraclass correlations suggest that, particularly for heroin use only and PO use only, a substantial portion of the total variability in type of use is attributable to variation between OTPs. Median odds ratios were 6.14, 5.18, and 2.41 for heroin only, PO only, and both, respectively. The median odds ratio considers the probability of use for two randomly chosen individuals from different OTPs. Conceptually, the odds ratio is formed by dividing the odds of use for the individual with a higher probability of use by the odds of use for the individual with a lower probability of use. Repeating this many times and calculating the median yields the median odds ratio, which quantifies variability between treatment programs on the common odds ratio scale. Particularly for heroin use only and PO use only, the median odds ratios are substantial and suggest that much of the total variability in type of use can be attributed to variation between OTPs.

Correlations between age and type of use. Table 2 shows point-biserial correlations between age and three types of opioid use. Between-OTP correlations are substantially larger than within-OTP correlations. This suggests the total association between age and each type of opioid use is primarily due to the association between the average age of enrolees in an OTP and the proportion of patients in an OTP with each pattern of use.

**Table 2:** Point Biserial Correlations Between Age and Type of Opioid use

	Within-OTP	Between-OTP
**Age with Heroin Only**	.07*	.69*
**Age with PO Only**	-.01	-.61*
**Age with Both Heroin & PO**	-.06*	.28*

Significance was determined using z-tests in which the covariance parameter estimate was divided by its standard error and compared with a critical value from the normal distribution (2.58, corresponding to *p *< .01). Covariances were converted to correlations for presentation here. Sample sizes for this analysis were 29114 individual enrolees nested within one of 85 OTPs.

* *p < .01*

Mixed multinomial logistic regression models. Table 3 shows the association of individual enrolee age with opioid use in a mixed multinomial logistic regression model. Enrolees 30-39, 40-49, and 50-59 years of age were less likely than younger enrolees to use PO only rather than heroin only. Enrolees 26-29, 30-39, 40-49, 50-59, and 60-70 years of age were less likely than younger enrolees to use heroin and PO rather than heroin only.

**Table 3:** Multinomial logistic multilevel model predicting type of opioid use: Helmert contrasts of individual age

	*PO & Heroin vs. Heroin Only*				*PO Only vs. Heroin Only*			
	Odds Ratio 95% Confidence Interval				Odds Ratio 95% Confidence Interval			
	Lower	Estimate	Upper	*p*	Lower	Estimate	Upper	*p*
**Individual Age Within Programs**								
20-25 vs. Younger	0.859	0.962	1.077	0.497	1.017	1.156	1.314	0.027
26-29 vs. Younger	0.873	0.915	0.959	<.001	0.930	0.980	1.033	0.446
30-39 vs. Younger	0.903	0.927	0.951	<.001	0.948	0.976	1.004	0.093
40-49 vs. Younger	0.913	0.931	0.948	<.001	0.944	0.964	0.986	0.001
50-59 vs. Younger	0.923	0.940	0.958	<.001	0.940	0.960	0.981	<.001
60-70 vs. Younger	0.888	0.926	0.965	<.001	0.927	0.973	1.021	0.269
**Interview Year**								
2006 vs. 2005	0.869	0.959	1.058	0.403	1.042	1.167	1.307	0.007
2007 vs. 2005	0.978	1.091	1.217	0.119	1.164	1.317	1.489	<.001
2008 vs. 2005	1.864	2.071	2.300	<.001	1.962	2.215	2.500	<.001
2009 vs. 2005	1.933	2.146	2.382	<.001	2.134	2.404	2.708	<.001
**Beale Urbanicity**								
Medium vs. High Density	1.883	2.130	2.410	<.001	2.783	3.155	3.578	<.001
Low vs. High Density	2.837	3.792	5.067	<.001	4.706	6.220	8.221	<.001
**U.S. Region**								
Southeast vs. Northeast	2.093	2.468	2.911	<.001	15.955	18.867	22.310	<.001
Midwest vs. Northeast	3.092	3.750	4.548	<.001	9.259	11.281	13.745	<.001
West vs. Northeast	0.899	0.997	1.105	0.954	2.241	2.526	2.846	<.001

**Note**: OTP averages of age contrasts were included in the model but are not shown here because it is not clear how to interpret them and the primary purpose of including them in the model is partialling out OTP-level age differences so that individual patient age contrasts can be unambiguously interpreted as pooled within-OTP associations. All associations were tested for significance using z-tests, in which the coefficient was divided by its standard error and compared with the normal distribution. Coefficients and confidence limits were exponentiated to odds ratios for presentation here. Sample sizes for this analysis were 29107 individual enrolees nested within one of 85 OTPs.

Enrolees completing the survey in more recent years were more likely to use both PO and heroin than heroin only and were more likely to use PO only than heroin only. Enrolees in programs located in areas with lower urbanicity were more likely to use both PO and heroin than heroin only and were more likely to use PO only than heroin only. Enrolees in programs located in the Southeast and Midwest regions of the U.S. were more likely than enrolees in programs located in the Northeast region of the U.S. to use both PO and heroin than heroin only and were more likely to use PO only than heroin only.

Table 4 shows the associations of OTP average age and individual enrolee age within OTP with opioid use in the month before OTP enrolment in a mixed multinomial logistic regression model. Relative to only heroin use, older individual enrolee age was associated with a reduction in the odds of PO use only and both PO and heroin use. Relative to only heroin use, older OTP average enrolee age was associated with a reduction in the odds of PO use only and both PO and heroin use.

**Table 4:** Multinomial logistic multilevel model predicting type of opioid use: Individual enrolee and OTP-average age

	*PO & Heroin vs. Heroin Only*				*PO Only vs. Heroin Only*			
	Odds Ratio 95% Confidence Interval				Odds Ratio 95% Confidence Interval			
	Lower	Estimate	Upper	*p*	Lower	Estimate	Upper	*p*
**OTP Average Age**	0.916	0.926	0.936	<.001	0.776	0.787	0.798	<.001
**Individual Age Within OTP**	0.979	0.982	0.985	<.001	0.984	0.988	0.991	<.001
**Interview Year**								
2006 vs. 2005	0.878	0.970	1.071	0.548	1.038	1.161	1.299	0.009
2007 vs. 2005	1.012	1.130	1.262	0.030	1.152	1.302	1.471	<.001
2008 vs. 2005	1.937	2.157	2.402	<.001	1.879	2.121	2.395	<.001
2009 vs. 2005	2.000	2.227	2.479	<.001	2.031	2.288	2.578	<.001
**Beale Urbanicity**								
Medium vs. High Density	2.280	2.536	2.821	<.001	3.439	3.837	4.281	<.001
Low vs. High Density	4.480	6.021	8.094	<.001	7.443	9.924	13.230	<.001
**U.S. Region**								
Southeast vs. Northeast	1.439	1.649	1.890	<.001	10.022	11.476	13.139	<.001
Midwest vs. Northeast	1.432	1.857	2.407	<.001	3.937	5.102	6.612	<.001
West vs. Northeast	0.794	0.865	0.942	0.001	1.931	2.141	2.374	<.001

**Note**: All associations were tested for significance using z-tests, in which the coefficient was divided by its standard error and compared with the normal distribution. Coefficients and confidence limits were exponentiated to odds ratios for presentation here. Sample sizes for this analysis were 29107 individual enrolees nested within one of 85 OTPs.

Enrolees completing the survey in more recent years were more likely to use PO and heroin than heroin only and were more likely to use PO only than heroin only. Enrolees in programs located in areas with lower urbanicity were more likely to use both PO and heroin than heroin only and were more likely to use PO only than heroin only. Enrolees in programs located in the Southeast and Midwest regions of the U.S. were more likely than enrolees in programs located in the Northeast region of the U.S. to use both PO and heroin than heroin only and were more likely to use PO only than heroin only. Enrolees in programs located in the West region of the U.S. were less likely than enrolees in programs located in the Northeast region of the U.S. to use both PO and heroin than heroin only and were more likely to use PO only than heroin only.

## Discussion

### Summary of findings

Patterns of opioid use varied considerably across OTPs. On the whole, while there were some significant associations between individual enrolee age and opioid use, effect sizes were small. Younger enrolees within OTPs had increased odds of using either POs only or both POs and heroin relative to heroin only. Effect sizes were larger for OTP average enrolee age, with heroin use more likely in OTPs with a higher average age and PO only or both PO and heroin use more likely in OTPs with a lower average age.

Enrolees interviewed in more recent years of the study period were more likely to use PO and heroin than heroin only and were more likely to use PO only than heroin only. Enrolees in OTPs located in higher urbanicity areas were more likely to use PO and heroin than heroin only and were more likely to use PO only than heroin only. Enrolees in OTPs located in the Midwest and Southeast regions of the U.S. were more likely than enrolees in OTPs located in the Northeast to use PO and heroin than heroin only and were more likely to use PO only than heroin only. Enrolees located in the West region of the U.S. were more likely than enrolees in OTPs located in the Northeast to use PO only than heroin only. Even after taking into account other factors including interview year, urbanicity, and U.S. region, younger OTP average age of enrolees was associated with an increase in the odds of PO use only and an increase in the odds both PO and heroin use.

### Discussion of findings

Consistent with previous research describing differences between heroin and PO users and addiction treatment admissions, we found increases in the probability of only heroin use and decreases in the probability of PO use, with or without heroin use, as individual enrolee age increased. These differences may be due in part to cohort effects, with younger users initiating drug use more recently when POs were more widely available.

While individual enrolee age differences consistent with previous research were found, it should be noted that effect sizes were fairly small. Contrasts of older age groups with younger age groups were typically associated with changes in the odds of a particular type of use that were around only 5%. While our main focus was on the association between individual enrolee age and type of use, we also found that variation in type of use across programs was substantial. This may simply reflect geographic differences in patterns of opioid use. The fact that the average age of enrolees in an OTP was related to opioid use more strongly than individual enrolee age suggests that both age and opioid use vary across OTPs and much of the total association between age and opioid use is at the OTP rather than the enrolee level. In other words, within a single OTP, individual enrolee age is only weakly associated with pattern of opioid use. Previous research examining differences among OTP enrolees with different patterns of use has not taken a multilevel approach. The multilevel approach allows individual enrolee characteristics and OTP characteristics to be disentangled, and we believe that is important for understanding the nature of individual differences in detail. If the nesting of enrolees within OTPs is ignored or if multilevel analysis is not formulated to differentiate associations at enrolee and OTP levels, then associations that primarily operate at the OTP level, and are thus organizational and cultural phenomena, may be misinterpreted as manifestations of the addiction process of the individual.

Many enrolees used heroin and POs in the month before enrolling in an OTP. This pattern of use may be an adaptation to changes in availability of heroin or prescription opioids. While previous research has suggested enrolees using POs generally have fewer problems than heroin users such as addiction severity, criminal activity and injection [3,4,20], it would be useful to examine enrolees who use a variety of opioids, including heroin, in more detail.

Consistent with a trend for an increase in first time treatment admissions for heroin among older adults [6], we found that heroin use was more prevalent among older OTP enrolees. We also found that prevalence of PO abuse among younger OTP enrolees is increasing over time, which is consistent with the dramatic increase in all PO treatment admissions for younger persons [5]. The increasing trends in PO abuse in the U.S. suggest that the OTP admissions for this disorder (especially among younger persons) will increase for the foreseeable future.

Since we also found that much of the total association between age and opioid use is at the OTP level rather than the enrolee level, we are likely to see that PO abuse will continue to remain more prevalent in some OTPs than in others. Collectively, a policy implication of these data is that OTPs will need to address the increasing prevalence of PO abuse among their newly enrolled patients and that some OTPs will continue to have a greater proportion of young PO abusing patients than others. Program service needs for young PO abusers compared with older heroin abusers are likely to differ, e.g., more vocational training for the younger and more chronic disease management for the older cohort. A one size fits all model would not be cost efficient. Given the increasing diversity among patients enrolling in opioid treatment (younger PO abusers; older heroin abusers) both within and especially across programs, OTPs will need to have some flexibility in how they utilize program resources. Moreover, policy makers and funders will need to be sensitive to these differences so that resources can be efficiently allocated.

### Limitations

Because OTPs were selected to represent U.S. regions where PO use was expected to be prevalent, the sample is not representative of the population of OTP enrolees. However, we do not believe this is a severe limitation. OTPs were sampled across diverse areas around the U.S., across 34 states and various geographical locations. More than half of the study OTPs were located in densely populated urban areas (where PO abuse is generally less prevalent).

## Conclusions

Organizational and cultural phenomena (e.g., OTP characteristics) must be examined to better understand the context of individual characteristics (e.g., age). If nesting of enrolees within OTPs is ignored, then associations that primarily operate at the OTP level may be misinterpreted as exclusively dependent on individual enrolees.

## Competing interests

The author declares that they have no competing interests.

## Authors' contributions

CMC designed and performed the statistical analysis and drafted the manuscript. AR participated in the design of the survey, supervised the collection of data, provided input on statistical analysis, and helped to draft the manuscript. CF managed the survey database and participated in the collection of data. CM participated in the design of the survey and the collection of data. All authors read and approved the final manuscript.
